# Auditory imagery and poetry-elicited emotions: a study on the hard of hearing

**DOI:** 10.3389/fpsyg.2025.1509793

**Published:** 2025-03-26

**Authors:** Simina Piţur, Ioana Tufar, Andrei C. Miu

**Affiliations:** ^1^Cognitive Neuroscience Laboratory, Department of Psychology, Babeş-Bolyai University, Cluj-Napoca, Romania; ^2^Department of Special Education, Babeş-Bolyai University, Cluj-Napoca, Romania

**Keywords:** poetry, emotions, auditory imagery, hearing loss, hard of hearing

## Abstract

Silent reading evokes auditory images of the written text, and there is emerging evidence that these images increase emotional arousal when reading poetry. A novel approach to studying their relevance to poetry-elicited emotions is to explore them in hard of hearing individuals, who may have difficulties generating mental images in this modality. In the present study, we investigated differences in auditory imagery, both as a dispositional trait and as a process that occurs during reading, and the intensity of poetry-elicited emotions between hard of hearing individuals and controls. We also explored whether the effect of hearing loss on arousal can be partially explained by the vividness of the auditory images evoked during reading. For this purpose, participants completed two sessions. First, they filled in a set of questionnaires concerning reading experience and dispositional traits. Second, they read poetry for 30 min, retrospectively rated their emotional responses to the poems and answered questions about socio-affective and cognitive processes during reading. Results showed that, although participants in the hard of hearing group scored significantly lower than controls on every measure of auditory imagery (i.e., trait auditory imagery, auditory imagery for words, and other sounds while reading), their emotions were no less intense. The hard of hearing group also reported lower levels of other dispositional traits (i.e., visual imagery and proneness to fantasizing), but not of any psychological processes during reading. Not much is known about the effects of mental imagery on poetry-elicited emotions, and our findings open a new and promising line of research for exploring their relevance and specificity.

## Introduction

Reading is good for you: what has long been considered a cliché is slowly gathering empirical support from various lines of research (Carney and Robertson, 2022; Dodell-Feder and Tamir, 2018; Kidd and Castano, 2013; Poerio and Totterdell, 2020), especially in relation to affective wellbeing. Literature offers rich social simulations for readers to engage in, which may diversify their daily emotional experiences, a mark of increased life satisfaction (Park et al., 2023); some of these emotions even have the potential to increase wellbeing almost immediately (i.e., awe; Monroy and Keltner, 2023). To understand how people can harness these benefits, it is important to examine how literature elicits emotions. Findings from empirical aesthetics can offer crucial insight into how people respond differently to verbal art and why.

Poetry makes an effective type of stimulus for studying literature in the laboratory, due to its brevity and emotional potency (“feeling special and powerful emotions” is the main reason why people report reading poetry; Piţur and Miu, 2022). Interest for this line of research is quite recent: studies are few and do not always approximate how poetry is read in day-to-day life (e.g., poems are recited; stimuli are few and homogeneous; Menninghaus et al., 2017; Obermeier et al., 2013). Nevertheless, there is emerging evidence that auditory imagery plays an important part poetry-elicited emotions (Piţur and Miu, 2022), namely that emotional arousal increases with the vividness of the “inner voice” acquired during silent reading for a variety of emotions (i.e., pleasing, negative, epistemic, and aesthetic).

It is important to note that individuals vary in their ability to imagine sound (Hinwar and Lambert, 2021). Certain events, such as early hearing loss, could disrupt the development of auditory imagery. Examining how poetry evokes emotions in such individuals may help estimate the affective costs of low auditory imagery, as well as the potential for other emotion-eliciting mechanisms (e.g., empathy, visual imagery) to compensate for them. Given that hearing loss is linked to lower generic quality of life (Nordvik et al., 2018), such findings may also help clarify whether reading could increase affective wellbeing in this population.

### Mental simulation in silent reading

Mental simulation is regarded as an important prerequisite for enjoying literature (Oatley, 2016). Often, the term is used synonymously with “mental imagery,” defined as accessing, combining, and modifying perceptual information in the absence of stimuli (Kosslyn et al., 2001). Although mental images can arise in many modalities during reading (e.g., Johnson et al., 2013), auditory images are probably generated the most consistently via translating orthographic information into phonological information (Leinenger, 2014). Despite ample evidence for complex vocal imagery in silent reading (Alexander and Nygaard, 2008; Gunraj and Klin, 2012; Hubbard, 2010), emotional responses to literature are rarely examined in relation to auditory imagery. This omission is especially surprising in the case of poetry, as one of its distinguishing features is the abundant use of sound similarities and recurrences (e.g., alliteration, assonance, consonance, rhyme, meter, etc.). If strongly patterned language translates into strongly patterned auditory images, poetry could engage mechanisms analogous to music to elicit emotions (Johnson-Laird and Oatley, 2022), in addition to empathizing with the authors or the characters (Oatley, 2016).

### Auditory imagery and poetry-elicited emotions

Johnson-Laird and Oatley (2022) make a strong case for the significance of auditory imagery in poetry-elicited emotions. They point out that poems sometimes describe sounds that can prompt auditory images (e.g., “Till human *voices* wake us, and we drown”; Eliot, 2009) and these are able to elicit emotions in and of themselves. More importantly, they argue that parallelistic features such as rhyme and meter, through mechanisms analogous to music, contribute independently to emotion elicitation. Indeed, experimentally altering these features significantly decreases the intensity of poetry-elicited emotions (Menninghaus et al., 2017; Obermeier et al., 2013).

One possible explanation for their emotional effects could be related to phonological recoding, the translation of orthographic information into phonological information that occurs during reading (Leinenger, 2014). This gives rise to what is sometimes described as the “inner voice”^1^: the subjective experience of hearing the words you are reading in a voice that may or may not be your own (Vilhauer, 2017). There seems to be considerable variability in the voices people hear when they read, both in terms of acoustic properties (e.g., pitch, duration, etc.) and emotional prosody (Vilhauer, 2017). Sometimes, the author (Alexander and Nygaard, 2008) and the characters (Kurby et al., 2009) are assigned distinct voices. In effect, it is a very specific form of auditory imagery, wherein a human voice is subjectively experienced in the absence of any auditory stimuli. Simply put, the assumption is that it is not the orthographic patterning of words that elicits emotions, but rather the acoustic patterning of sounds they generate in the reader's mind. To conclude, out of all imagery modalities, auditory images (specifically, auditory images of words) might be elicited the most consistently during reading by virtue of the phonological recoding process. When and how often visual images are evoked depend on authorial choices: one poet may aim for creating rich descriptions of objects, places or people, while another might be more concerned with exploring complex ideas. In contrast, words of any poem can be translated into their corresponding auditory images.

To our knowledge, only one study has investigated the link between auditory imagery and poetry-elicited emotions (Piţur and Miu, 2022). Here, imagery was measured both as a dispositional trait (the general ability to generate and control auditory images) and as a cognitive process that occurs during reading (i.e., participants were asked to what extent they “heard” the words of the poems they were reading in their mind). Results showed that arousal increased with higher trait and process auditory imagery, but also hinted at long-term interactions between reader characteristics. Those who had read more poetry in the past heard the words more vividly in their mind, and this effect was stronger for readers with better trait auditory imagery. In short, an overall good ability to generate and manipulate imaginary sounds seemed to offer an important advantage: it potentiated the long-term effects of reading experience on the vividness of imagined words, which in turn lead to more intense emotions.

### Hearing loss and auditory imagery

Hearing loss might lead to atypical auditory imagery through affecting auditory perception. Neuroimaging data lend some support to this assumption, given some evidence for common neural mechanisms: the brain areas involved in the perception and the imagining of sound overlap partially (for a review, see Hubbard, 2010). Early auditory deprivation has been linked to several structural and functional changes in these areas (for a review, see Kral, 2013), most notably cross-modal reorganization (e.g., the auditory cortex is recruited for processing visual stimuli in early-deafened individuals; Bola et al., 2017; Fine et al., 2005; Finney et al., 2001) and it is possible that these changes also alter the generation of auditory imagery. Moreover, early hearing loss appears to alter the development of phonological awareness (Mayer and Trezek, 2014) and the processing of parallelistic features such as rhyme (Sterne and Goswami, 2000). This hints at a possible atypical development of auditory imagery for verbal content in the hard of hearing.

Nevertheless, only a handful of studies have investigated the link between auditory deprivation and auditory imagery (Heinen et al., 1976; Le Craft, 1935). Given several important methodological limitations, their results are difficult to interpret and generalize. For instance, while Heinen et al. (1976) found that the congenitally deaf individuals in their study struggle to learn word pairs that are presumed to evoke auditory imagery (e.g., “music-scream,” “whisper-explosion”), they did not measure imagery *per se* and could neither confirm auditory images were evoked, nor assess their vividness. Standardized assessments of auditory imagery could help clarify this relation, as well as considering wider ranges of hearing loss.

### The present study

Provided that sensory deprivation impedes the development of auditory imagery, could it also make individuals with hearing loss less susceptible to the emotional effects of poetry? The present study examines whether, granted that hard of hearing individuals report difficulties in imagining sound, in general, and hearing the words they are reading, in particular, they also report dampened poetry-elicited emotions. To this end, our main aim was to investigate differences in auditory imagery and poetry-elicited emotions between hard of hearing and hearing individuals. Using a similar procedure as the aforementioned study (Piţur and Miu, 2022), participants completed a series of questionnaires, read poems for 30 min, subsequently rated the intensity of their emotions and answered a few questions about their experiences during reading. First, we expected the hard of hearing group to report lower levels of trait auditory imagery, and less vivid auditory images of words and other sounds while reading. Second, given the link between auditory imagery and poetry-elicited emotions (Piţur and Miu, 2022), and considering the possibility that phonological knowledge deficits associated with hearing loss alter the processing of parallelistic features, we also expected the hard of hearing group to report less intense emotions during reading.

Which mechanisms might explain the impact of hearing loss on poetry-elicited emotions? A secondary aim was to investigate a possible mediator role of auditory imagery. Given that the vividness of the words readers hear in their mind has been found to predict the intensity of poetry-elicited emotions (Piţur and Miu, 2022), we hypothesized that auditory imagery for words during reading would be a mediator in the relation between hearing loss on arousal.

Finally, we explored links between hearing loss and other dispositional traits (trait empathy, visual and movement imagery, and proneness to fantasizing) and psychological processes (empathy for author and characters, vividness of visual and movement imagery during reading) relevant to poetry-elicited emotions (Piţur and Miu, 2022). Such links could offer insight into possible complementary deficits or compensatory mechanisms. We also looked at different aspects of hearing loss (diagnosis, rehabilitation, and communication preferences) to find more specific associations with arousal and auditory imagery.

## Materials and methods

### Participants

The study was advertised through flyers placed in hearing-aid stores and through Facebook Ads and vouchers for several shops were offered as rewards. Twenty-one participants were assigned in the hard of hearing group based on their answers to questions regarding their diagnosis; only individuals who had been officially diagnosed with hearing loss and self-reported good reading and writing abilities were included. Twenty-one controls, matched for sex, age, and education, were selected from a large pool of healthy recruited participants. In total, 42 participants (85.71% women), aged between 17 and 73 (*M* = 34.07; *SD* = 17.63), completed the study on-line. We used previously developed Romanian translations of all questionnaires (Piţur and Miu, 2022), with good psychometric properties.

### Procedure

**Figure 1:**
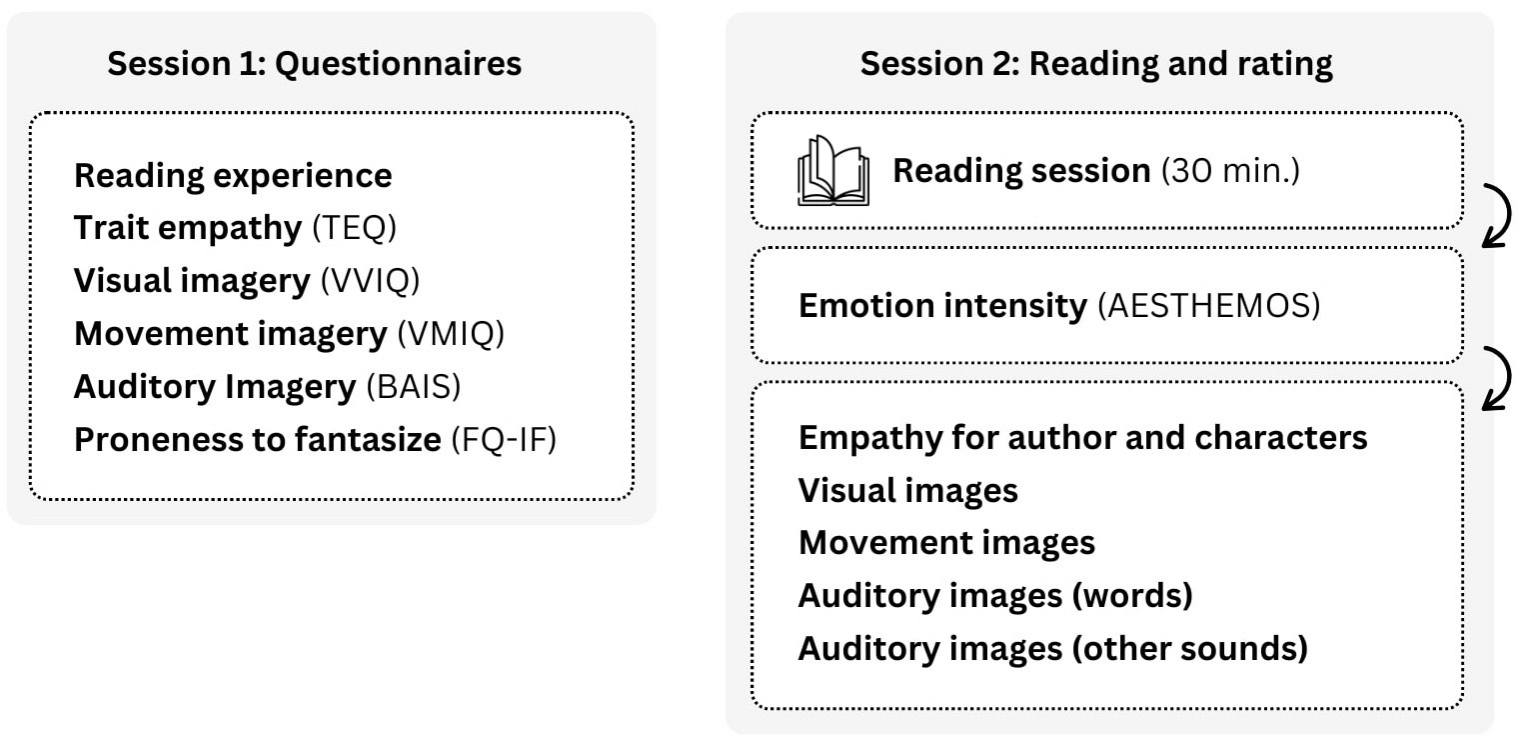
Procedure. TEQ, Toronto Empathy Questionnaire; VVIQ, Vividness of Visual Imagery Questionnaire; VMIQ, Vividness of Movement Imagery Questionnaire; BAIS-V, Bucknell Auditory Imagery for Sounds, Vividness subscale; BAIS-C, Bucknell Auditory Imagery for Sounds, Control subscale; FQ-IF, Fantasy Questionnaire, Imaginative Fantasy subscale; AESTHEMOS, The Aesthetic Emotions Scale.

To minimize fatigue, the study was split into two equal-length sessions that participants completed within 2 weeks. First, they filled reading experience and dispositional traits questionnaires (i.e., trait empathy, trait visual imagery, trait movement imagery, trait auditory imagery, and proneness to fantasize). Second, they were asked to spend 30 min reading from a set of poems made available by the researchers. They were told they could read any of the poems, in any order, and were provided with an interactive table of contents to facilitate their navigation through the large collection. The set was created and used in a previous study on Romanian participants to elicit a wide range of emotions (Piţur and Miu, 2022), from anthologies covering a wide range of genres over the course of several centuries (for more details, see Supplementary Table 1). After the reading session, participants retrospectively rated the intensity of the emotions they felt during reading. They also answered a few other questions about psychological processes during reading: to what extent they empathized with the author and characters, and to what extent they experienced visual images, motor images, auditory images for words, and auditory images for other sounds. For hard of hearing participants, we also collected data about their diagnosis, hearing aids or cochlear implants, and preferences for using sign language or lip reading, in the interest of exploring their associations with auditory imagery and poetry-elicited emotions (Figure 1).

### Measures

#### Hearing loss

Participants indicated when they had started to lose their hearing: before turning 1 and a half, between 1 and a half and 10, and between 11 and 19, 20 or older. They were also asked to mention diagnosis (conduction loss, sensorineural loss or mixed), severity (mild, moderate, severe, or profound), and laterality (unilateral or bilateral). If they mentioned using a hearing aid or cochlear implant, we inquired about when they had started wearing it and its amplification level (low, medium, high). We also examined if participants use lip reading and sign language in their day-to-day life, and whether their friends and parents use sign language. Lastly, we asked whether at least one of their parents had been diagnosed with hearing loss.

#### Reader characteristics

##### Reading experience

We measured reading experience by asking a few questions about participants' reading history and habits (Piţur and Miu, 2022). First, they indicated what they considered to be the longest period of having read poetry on their own initiative (“none,” “several days,” “several months” or “several years”) and the age at which this period occurred. For our descriptive analyses, we recoded their answers into the following categories: childhood (before age 10), adolescence (age 11–19), and adulthood (age 20 and older). For our main analyses, the continuous measure of age was used. Second, they indicated how often they had read poetry during the last 6 months (“never/almost never,” “once every few months,” “each month,” and “each week”).

##### Dispositional traits

Trait auditory imagery was assessed with the Bucknell Auditory Imagery Scale (BAIS; Halpern, 2015). BAIS items cover two aspects of auditory imagery: vividness and control. Respondents are asked to imagine certain sounds (e.g., the sound of a gentle rain) and to rate how vivid the auditory images are and how easy it is to change them (e.g., the rain turns into a violent thunderstorm). Total scores for vividness and controllability were created and used in all analyses, ranging from 14 to 98. The questionnaire showed excellent validity for both the auditory vividness subscale (Cronbach's alpha = 0.93, 14 items) and the auditory control subscale (Cronbach's alpha = 0.94, 14 items). High scores reflect high imagery ability.

Proneness to fantasizing was measured with the Imaginative Fantasy subscale of the Fantasy Questionnaire (FQ-IF; Weibel et al., 2018), which asks respondents about fantasies, daydreams and mind-wandering (e.g., “my daydreams are often stimulating and rewarding”). The FQ-IF showed good validity (Cronbach's alpha = 0.83, 16 items) in the present sample. A total score, ranging from 16 to 80, was created and used in analyses. Higher values indicate higher proneness to fantasizing.

Trait empathy, the accurate affective insight into the feeling state of another, was measured with the Toronto Empathy Questionnaire (TEQ; Spreng et al., 2009). The TEQ includes items pertaining to emotional contagion, emotion comprehension, sympathetic physiological arousal, and conspecific altruism. The questionnaire showed good validity (Cronbach's alpha = 0.80, 16 items) in the present sample. A total TEQ empathy with possible values ranging from 0 to 64 was used in the analyses. Higher scores reflect higher trait empathy.

Trait visual imagery was measured with the Visual Vividness of Imagery Questionnaire (VVIQ; Marks, 1973). The VVIQ presents vignettes describing visual scenes (e.g., a sunrise) to which new elements are added (e.g., a rainbow appears). Participants rate the vividness of these scenes as they imagine them. The questionnaire showed excellent validity (Cronbach's alpha = 0.90, 16 items) in the present sample. A total VVIQ visual imagery score, ranging from 16 to 80, was used in the analyses. Higher scores reflect higher trait visual imagery.

Trait movement imagery was measured with the revised version of the Vividness of Movement Imagery Questionnaire (VMIQ-2; Roberts et al., 2008). The VMIQ prompts participants to imagine executing a set of movements (e.g., throwing a rock) from an internal perspective (i.e., as if they are looking through their own eyes), from an external perspective (i.e., as if they are watching themselves performing the movement), and to imagine what doing the movement feels like. The VMIQ showed excellent validity (Cronbach's alpha = 0.97, 36 items) in the present sample. Separate scores for the three subscales were created by summing vividness ratings. A total VMIQ movement imagery score, ranging from 38 to 180, was then created from the sum of the three and used in subsequent analyses. Higher scores indicate higher trait movement imagery.

#### Poetry-elicited emotions

We used the Aesthetic Emotions Scale (AESTHEMOS; Schindler et al., 2017) to assess participants' emotional responses to the poems. Participants rated the extent to which they felt each of the 21 discrete emotions described in the questionnaire, covering four broad categories: aesthetic emotions (e.g., being moved), pleasing emotions (e.g., joy), epistemic emotions (e.g., interest), and negative emotions (e.g., sadness). Separate scores for each emotion category (i.e., aesthetic, pleasing, epistemic, and negative) were calculated for descriptive analyses. A total AESTHEMOS score was created as an indicator of emotional arousal in our analyses.

#### Psychological processes during reading

We asked participants several questions about empathy and imagery during reading (for more details about item creation and scoring, see Piţur and Miu, 2022). They rated to what extent they experienced poetry-related empathy and how vivid their visual imagery, movement imagery, auditory imagery for words, and auditory imagery for other sounds had been during reading.

#### Socio-economic status

Education level was classified into (1) middle school level and lower, (2) high school level, and (3) undergraduate level and higher. Occupation was classified into 10 major groups, as specified by the International Standard Classification of Occupations of the International Labour Organization (ISCO-88; Elias and Birch, 1988).

###  Statistical analyses

Characteristics of the present sample were first summarized with descriptive statistics. We then explored correlations between reader characteristics, psychological processes during reading, and emotions in the entire sample. For our confirmatory analyses, we first compared reader characteristics, psychological processes, and poetry-elicited emotions between the two groups using independent two-sample *t*-tests. Then, we investigated the mechanistic role of auditory imagery for words: we estimated a causal mediation effect, having fitted a model for the conditional distribution of auditory imagery for words given hearing loss, and a model for the conditional distribution of arousal given auditory imagery for words and hearing loss. For our planned exploratory analyses, we further used *t*-tests and correlation analyses to investigate links between hearing loss and other reader traits and psychological processes, and between certain aspects of hearing loss, arousal, and auditory imagery.

All analyses were carried out in R (R Developement Core Team, 2013), using the *mediation* package for causal mediation analysis (Tingley et al., 2014). The database and R code can be downloaded here:https://osf.io/za2cg/?view_only=ed2231e7b9434b48b4f76fdb131aed51.

## Results

### Descriptive analyses

In the hard of hearing group, most participants received their first diagnosis in childhood, with more than half reporting a current diagnosis of sensorineural hearing loss and profound severity; all but one were affected bilaterally (see Table 1). Only a few participants reported not using a hearing aid and lip reading was preferred over signing in day-to-day life (see Table 1). There were some notable differences between the hard of hearing and the control group regarding reading experience (see Table 2), indicating that hard of hearing participants had read poetry less often. Descriptive statistics for dispositional traits, psychological processes during reading, and arousal for all AESTHEMOS emotion categories (i.e., aesthetic, pleasing, epistemic, and negative) are presented in Table 3.

**Table 1:** Hearing loss.

		**Percentage %**
Age of onset	< 18 months	33.33
	18 months to 10 years	33.33
	11 years to 19 years	14.29
	20 years or older	19.05
Diagnosis	Conduction	4.76
	Sensorineural	57.14
	Mixed	4.76
	Not sure	33.33
Severity	Mild	14.29
	Moderate	14.29
	Severe	19.05
	Profound	52.38
Laterality	Unilateral	4.76
	Bilateral	90.48
Hearing aid (first use)	< 18 months	0
	19 months to 10 years	42.86
	11 years to 19 years	9.52
	20 years or older	33.3 3
Hearing aid (current use)	No hearing aid	19.05
	Low amplification	19.05
	Medium amplification	19.05
	High amplification	42.85
Amplification power (current use)	Not applicable (no hearing aid)	19.05
	Low	19.05
	Medium	19.05
	High	42.85
Communication	Signing	19.05
	Lip reading	80.95
	Parent diagnosed with hearing loss	9.52
	Signing parent	9.52
	Signing friends	57.14

All percentages are calculated from the total number of participants in the hearing-impaired group.

**Table 2:** Demographic data and reading experience.

		**Hard of hearing**	**Control**
Education	Middle school or lower	4.76%	4.76%
	High school	26.19%	26.19%
	Undergraduate or higher	69.48%	69.48%
Occupation	Group 1: Legislators, senior officials, and managers	0%	4.76%
	Group 2: Professionals	47.62%	52.38%
	Group 3: Technicians and associate professionals	9.52%	0%
	Group 4: Clerks	9.52%	4.76%
	No occupation	33.33%	38.10%
Longest period reading poetry	A few days	42.86%	38.10%
	A few months	28.57%	4.76%
	A few years	23.81%	52.38%
	None	4.76%	4.76%
Age at which reading poetry began	Childhood	14.29%	0%
	Adolescence	41.43%	80.95%
	Adulthood	9.52%	4.76%
	None	4.76%	14.28%
Reading habits over the last 6 months	Every week	23.81%	14.29%
	Every month	9.52%	23.81%
	Every few months	19.05%	38.10%
	None	47.62%	23.81%

As education was one of the matching criteria for selecting healthy participants, each educational level was equally represented in both the hearing impaired and control groups. Most participants (66.66%) who reported no occupation also specified they were students.

**Table 3:** Reader traits, socio-affective and cognitive processes, and emotions.

	**Hard of hearing**				**Control**				***t*(39)**	***p*-value**	**Cohen's *d***
	**Min**	**Max**	***M***	***SD***	**Min**	**Max**	***M***	***SD***			
**Reader traits**											
1. Trait empathy (TEQ)	33	60	46.38	6.72	31	61	48.86	8.13	1.00	0.3	0.33
2. Visual imagery (VVIQ)	18	76	55.05	11.54	48	78	65	8.81	3.00	0.003	0.97
3. Movement imagery (VMIQ)	36	179	117	34.94	50	140	113.4	21.11	−0.4	0.07	0.13
4. Auditory imagery vividness (BAIS-V)	1	6.7	3.73	1.63	3	6.8	5.31	1.09	4.46	0.001	1.14
5. Auditory imagery control (BAIS-C)	4.12	6.64	3.99	1.55	4.21	6.71	5.68	0.78	4.47	0.001	1.38
6. Proneness to fantasizing (FQ-IF)	33	63	50.52	9.47	36	74	59.05	11.47	3.00	0.01	0.81
**Processes**											
1. Empathy for authors and characters during reading	4	19	12.43	4.07	6	19	14.43	3.54	2.00	0.01	0.52
2. Visual imagery during reading	1	5	3.14	1.15	2	5	3.76	0.89	2.00	0.06	0.6
3. Movement imagery during reading	5	8	10.76	3.24	5	8	9.95	2.85	−0.09	0.04	0.27
4. Auditory imagery for sounds during reading	1	7	2.48	1.99	1	5	3.43	1.47	2.00	0.09	0.54
5. Auditory imagery for words during reading	1	6	2.86	1.74	1	4	4.33	1.53	3.00	0.006	0.9
**Emotion intensity**											
1. Aesthetic emotions	18	58	38.71	12.09	23	58	42.33	8.74	1.00	0.03	0.34
2. Pleasing emotions	11	50	27.67	9.88	12	39	28.19	7.51	0.2	0.08	0.06
3. Epistemic emotions	16	35	26.14	12.09	18	36	27.71	4.85	1.00	0.03	0.3
4. Negative emotions	14	38	24.33	7.58	12	34	22.19	5.68	−1.00	0.03	0.32
5. Total	75	158	116.9	23.23	75	151	120.4	17.62	0.06	0.06	0.17

TEQ, Toronto Empathy Questionnaire; VVIQ, Vividness of Visual Imagery Questionnaire; VMIQ, Vividness of Movement Imagery Questionnaire; BAIS-V, Bucknell Auditory Imagery for Sounds, Vividness subscale; BAIS-C, Bucknell Auditory Imagery for Sounds, Control subscale; FQ-IF, Fantasy Questionnaire, Imaginative Fantasy subscale.

### Auditory imagery and poetry-elicited emotions

Our principal aim was to investigate differences in auditory imagery and poetry-elicited emotions between the two groups. As hypothesized, we found that hard of hearing participants reported lower levels of both trait and process imagery. They scored significantly lower on both subscales of trait imagery [BAIS-V: *t*_(34.92)_ = 3.69, *p* < 0.001; BAIS-C: *t*_(29.57)_ = 4.47, *p* < 0.001] than controls [BAIS-V: *M* = 5.31, *SD* = 1.09; BAIS-C: *M* = 5.68, *SD* = 0.78]. When asked about auditory images evoked during the reading session, they reported hearing words less vividly in their mind [*M* = 2.86, *SD* = 1.74, *t*_(39.34)_ = 2.92, *p* = 0.005] than controls (*M* = 4.33, *SD* = 1.53), but not other types of sound [*t*_(36.80)_ = 1.76, *p* = 0.086]. Contrary to our expectations, we did not find any significant differences in the intensity of poetry-elicited emotions between hard of hearing and control participants [*t*_(37.29)_ = 0.56, *p* = 0.578].

**Figure 2:**
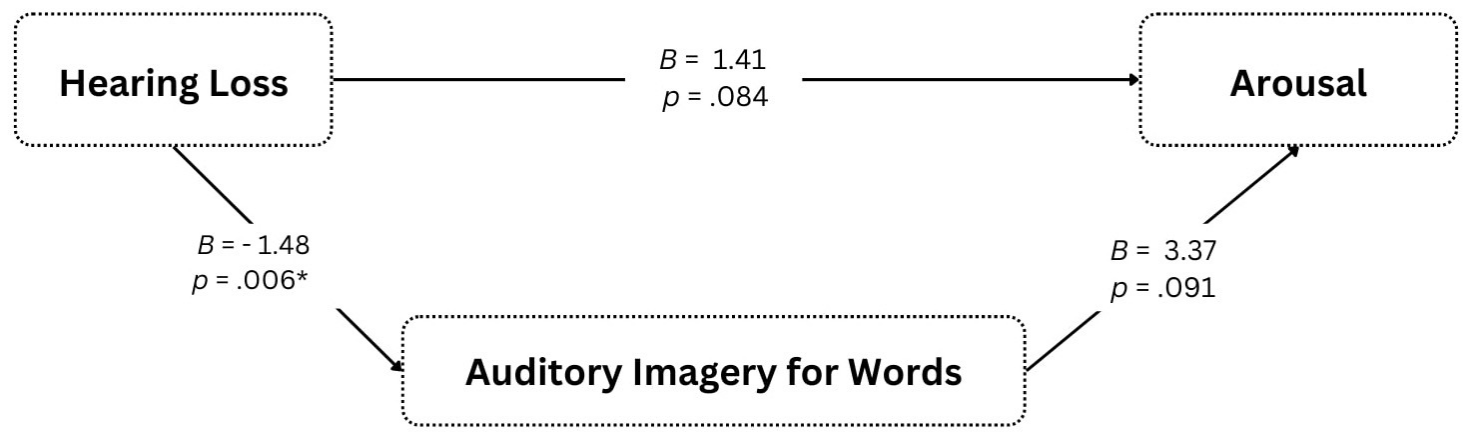
Mediation analysis. Mediation analysis testing the hypothesis that auditory imagery for words during reading mediates the relation between hearing loss and arousal of poetry-elicited emotions. *p < 0.05.

A secondary aim was to explore relations between hearing loss, auditory imagery, and arousal. Although a significant, negative association was found between hearing loss and auditory imagery for words during reading (*B* = – 1.48, *p* = 0.006), the latter did not predict arousal and was not a significant mediator (see Figure 2). Contrary to our hypothesis, the weaker auditory images of words in the hard of hearing did not impact the intensity of their emotions.

### Exploratory analyses

We further examined other possible differences regarding dispositional traits and cognitive mechanisms between the two groups. The hard of hearing group reported significantly lower levels of trait visual imagery [*t*_(37.41)_ = 3.14, *p* = 0.003], compared with the control group, and significantly lower levels of proneness to fantasize [*t*_(38.62)_ = 2.63, *p* = 0.012]. No significant differences were found regarding trait empathy and trait movement imagery, nor regarding processes empathy, visual or movement imagery during reading. Mediation analyses were ran to investigate mechanisms alternative to auditory imagery for words: neither poetry-related empathy, nor visual imagery, nor movement imagery mediated the relation between hearing loss and arousal (all *p*s > 0.05).

It is noteworthy that, apart from trait movement imagery, all reader traits showed significant, moderate and positive correlations with their homologous processes: trait empathy with empathy for authors and characters, trait visual imagery with visual images while reading, and trait auditory imagery with auditory imagery for words and other sounds while reading.

Lastly, we explored possible links between specific aspects of hearing loss and arousal, on the one hand, and auditory imagery, on the other. First, lower arousal was associated with later adoption of correction devices: the age at which participants started using a hearing aid correlated negatively with the intensity of poetry-elicited emotions (*rho* = −0.48, *p* = 0.040). Second, weaker auditory imagery was linked to certain communication preferences. Trait auditory imagery scores were lower for participants who use sign language in social interactions [BAIS-V; *M* = 2.08, *SD* = 1.32, *t*_(4.927)_ = 2.72, *p* = 0.042] than not (BAIS-V; *M* = 4.12, *SD* = 1.47), and for those with at least one signing parent [BAIS-V: *M* = 1.50*, SD* = 0.71, *t*_(2.189)_ = 4.04, *p* = 0.048; BAIS-C: *M* = 1.179, *SD* = 0.25, *t*_(10.085)_ = 8.91, *p* < 0.001] than none (BAIS-V: *M* = 3.96*, SD* = 1.52; BAIS-C: *M* = 4.28*, SD* = 1.30). During reading, auditory imagery for words was less vivid for participants who use lip reading in social interactions [*M* = 2.35, *SD* = 1.50, *t*_(8.616)_ = 4.84, *p* = 0.001] than not (*M* = 5.00, *SD* = 0.82).

## Discussion

The present study investigated whether hard of hearing individuals present atypical auditory imagery and experience poetry-elicited emotions differently. As hypothesized, the hard of hearing group reported lower levels of trait auditory imagery and less vivid auditory imagery for words during reading. However, the emotions they felt during reading were not significantly less intense than those of hearing participants.

To our knowledge, these results are the first to show that, despite known risks for a detrimental effect on language development (Duchesne, 2016; Fagan, 2016; Most, 2016), hearing loss does not make individuals significantly less receptive to the emotional effects of poetry. We put forth several explanations for these results. It is possible that the different reading strategies employed by individuals with hearing loss (Mayer and Trezek, 2014) foster emotional effects through different mechanisms. Neurophysiological data suggest that equally skilled deaf and hearing readers process text in different ways (Mehravari et al., 2017) and some research indicates that good grapheme-phoneme conversion skills can be developed in spite of weaker phonological awareness (Gravenstede, 2009). Although hard of hearing participants in our sample did not report greater empathy or more vivid imagery in other modalities (in fact, we found an opposite pattern for trait visual imagery), these effects might have been too small to detect in our small sample and, in addition, other compensatory mechanisms could also be involved and worth investigating in the future. Moreover, early rehabilitation might buffer against some of the effects of hearing loss: although hard of hearing participants, overall, did not report less intense emotions than controls, those who began using correction devices at a later age reported lower arousal. On the other hand, even if they fall behind hearing individuals in some aspects of language processing, the skills they do possess might be enough to let the emotional effects of poetry unfold: for instance, although deaf children's abilities to make rhyme judgements is less accurate than that of hearing children (Sterne and Goswami, 2000), they still perform above chance. We believe these findings should be particularly encouraging for special educators who are considering using poetry in the classroom.

Exploring which characteristics of hearing loss were associated with lower levels of auditory imagery, we found several small and seemingly contradicting associations with communication preferences: signing was linked to lower trait auditory imagery, but lip-reading was linked to lower auditory imagery for words during reading. Since most participants reported lip-reading and not signing in day-to-day life, a more heterogeneous sample would be needed to clarify the relations between auditory imagery and the two. However, we believe there could be one plausible explanation for the latter finding: if visual cues are used more than auditory cues in lip-reading, it is possible that auditory representations of words weaken in time. Future studies could also investigate if specific abilities are affected by hearing loss and impact auditory imagery for words, such as phonological awareness.

There are several limitations to the generalizability of our results. First, there is the issue of our small sample size and low heterogeneity of a few variables (i.e., socio-economic status, diagnosis, rehabilitation, and communication preferences). Most participants had undergraduate or higher levels of education and were professionals; a recruitment bias perhaps determined by our choice of placing flyers in hearing-aid stores, which inadvertently selected participants on the higher end of financial income. Second, in the interest of reducing participant fatigue, we did not control for the actual time spent in-task, order effects or selection biases, all of which may have consequences on emotion. However, data from a previous study using the same collection of poems (Piţur and Miu, 2022) showed that most participants were able to read for the allotted time without interruptions, and varied both in their choices of poems, and the order in which they read them. Third, we did not measure any text characteristics, hence we cannot estimate to what extent levels of poetry-related empathy, visual imagery, or motor imagery depended on characteristics of the reader rather than on the content of the poems. If developed in the future, datasets providing such information, as well as normative emotion ratings, would prove extremely valuable to disentangling the effects of text and reader characteristics on poetry-elicited emotions. Lastly, to avoid recall biases, measuring emotions after each poem rather than at the end of a reading session in future studies would be a much-needed methodological improvement. This would also allow for a multilevel modeling approach with items (i.e., poems) as a random factor, better suited to investigate if results could be generalized to different sets of poems.

A particularly interesting result of our exploratory analyses is that, although hard of hearing participants reported lower levels of trait visual imagery than controls, the visual images evoked by the poems were, in fact, comparably vivid. Although individual differences are useful in explaining some of the variance of arousal (Piţur and Miu, 2022), measuring what actually happens during reading is important; the moderate correlation between traits and their homologous processes might indicate that other factors (e.g., working memory, motivation, and mood) influence the implementation of a general ability in a particular context, and must be accounted for. Furthermore, an important issue is whether the ability to generate mental images is multimodal or modality-specific. Although a good ability to generate mental images in one modality does not guarantee similar abilities in another (Andrade et al., 2014), many people do experience multimodal deficits (Dawes et al., 2024). The positive associations found in our study between trait auditory and visual imagery, on the one hand, and between both types of imagery and proneness to fantasizing, suggest there are common mechanisms that could account for individual differences regarding not one, but many imagery modalities.

Finally, assessing verbal imagery, as opposed to generic auditory imagery, could provide more fine-grained insights. Auditory simulations of a poem may elicit emotions via two routes. The first is self-sufficient: prosody can directly induce emotions through emotional contagion. The second, however, implies that one mechanism engages another: the auditory simulation of the “voice” is integrated into multimodal simulations of the author or characters in the poem, informing the reader's empathy, sympathy or antipathy. Here, dimensions other than vividness may be relevant, dimensions that are specific to speech: whether the reader hears their own voice or assigns distinctive voices to different characters, and whether they express emotion. To the authors' knowledge, the few instruments that measure verbal imagery target mostly spontaneous inner speech and self-talk (e.g., Alderson-Day et al., 2018). Future studies may need to adapt such measures to specifically assess the ability to transform a written text into an “inner voice”.

## Conclusions

The present study has shown that hard of hearing individuals experience similar poetry-elicited emotions to hearing controls, despite significantly lower trait auditory imagery, and auditory imagery for words during reading. These findings suggest that, in hard of hearing individuals, emotion elicitation relies more on other mechanisms, compensating for weaker auditory imagery. Larger and more heterogeneous samples, more granular measurements of emotions, and controlling for multiple reader and text characteristics are needed to clarify these relations. We argue that exploring how poetry evokes emotions in populations with atypical imagery abilities is a promising new line of research, especially useful in uncovering when and why certain eliciting mechanisms are engaged, relative to others.

## Data Availability

The datasets presented in this study can be found in online repositories. This data can be found here: https://osf.io/za2cg/?view_only=ed2231e7b9434b48b4f76fdb131aed51.

## Footnotes

^1^There is some debate about whether subvocalization includes articulatory as well as auditory imagery (see Hubbard, 2010). For the purpose of this article, we use the term “inner voice” to describe auditory imagery only.
