# Supplementary material for: Auditory imagery and poetry-elicited emotions: a study on the hard of hearing
Source: Front Psychol. 2025 Mar 26;16:1509793. doi: 10.3389/fpsyg.2025.1509793 (PMC11979220; doi:10.3389/fpsyg.2025.1509793)
Supplement: Supplementary file 1 [file Table_1.docx]

**Supplementary Table 1.** *Poems*

|  | **Title** | **Author** | **Translator** |
| --- | --- | --- | --- |
| 1 | *N–am vrut* | Federico Garcia Lorca | Darie Novăceanu |
| 2 | *Ninge cu dușmănie* | Ana Blandiana | - |
| 3 | *Nimica toată* | Guillaume Apollinaire | Mihai Beniuc |
| 4 | *Vaza* | Shinabu Itomi | Marin Sorescu |
| 5 | *De-ar fi mijloace* | Mihai Eminescu | - |
| 6 | *Despărțire* | W. S. Merwin | Marin Sorescu |
| 7 | *Ca să faci portretul unei păsări* | Jacques Prévert | Gellu Naum |
| 8 | *Să mă prezint puțin* | Robert Șerban | - |
| 9 | *Nasturele* | Julian Kornhauser | Constantin Geambașu |
| 10 | *Nu există* | Nichita Stănescu | – |
| 11 | *Și pe dată e seară* | Salvatore Quasimodo | Mihai Banciu |
| 12 | *Și totuși* | Vladimir Maiakovski | Leo Butnaru |
| 13 | *Contemporan* | Vasile Voiculescu | - |
| 14 | *Danse russe* | William Carlos Williams | Mircea Ivănescu |
| 15 | *Sonet CLVII* | Vasile Voiculescu | – |
| 16 | *Călătoria cu Stelică* | Gellu Naum | – |
| 17 | *Cel zidit* | Giorgio Caproni | Aurora Firța |
| 18 | *Ca să fii mare, fii întreg* | Fernando Pessoa | Dinu Flămând |
| 19 | *Scoica* | Lucian Blaga | – |
| 20 | *Speranța e ceva cu pene* | Emily Dickinson | Veronica Porumbacu |
| 21 | *A se citi dimineața și seara* | Bertolt Brecht | Nina Cassian |
| 22 | *Sunt tânăr, doamnă* | Mircea Dinescu | – |
| 23 | *A se citi dimineața și seara* | Bertolt Brecht | Nina Cassian |
| 24 | *Monorima* | Gheorghe Tomozei | – |
| 25 | *Frig* | Ion Mureșan | – |
| 26 | *Ozymandias* | Percy Bysshe Shelley | Petre Solomon |
| 27 | *Cal aș fi vrut să fiu* | Nora Iuga | – |
| 28 | *Câtecul de dragoste al lui J. Alfred Prufrock* | T. S. Eliot | Mircea Ivănescu |
| 29 | *Jucării* | Marin Sorescu | – |
| 30 | *Poezia cu greșeală de tipar* | Oskar Pastior | Corina Bernic |
| 31 | *Acuma că sunt îndrăgostit* | Fernando Pessoa | Dinu Flămând |
| 32 | *Portret* | Emil Botta | – |
| 33 | *Văd niște nebuni* | Tadeusz Różewicz | Constantin Geambașu |
| 34 | *Annabel Lee* | Edgar Allan Poe | Lucian Blaga |
| 35 | *Despre tata și noi* | Cristian Popescu | – |
| 36 | *La florăreasă* | Jacques Prévert | Gellu Naum |
| 37 | *Satul teton* | Allen Ginsberg | Marin Sorescu |
| 38 | *Luasem forma unui fluture* | Ileana Mălăncioiu | – |
| 39 | *Sonet 154* | William Shakespeare | Gheorghe Tomozei |
| 40 | *Zădărnicie* | Giuseppe Ungaretti | Ilie Constantin |
| 41 | *Poezia sau viața* | Andrei Bodiu | – |
| 42 | *Mic dejun pentru doi streini* | Carl Sandburg | George Macovescu |
| 43 | *M–a mângâiat o fantomă* | Walt Whitman | George Macovescu |
| 44 | *Dezîmblânzirea* | Nichita Stănescu | – |
| 45 | *Tu nu știi colinele…* | Cesare Pavese | Nicolae Argintescu–Amza |
| 46 | *Triunghiul* | Gellu Naum | – |
| 47 | *Treisprezece chipuri de a privi o mierlă* | Wallace Stevens | Leon Levițchi |
| 48 | *În timp ce eu* | Nina Cassian | – |
| 49 | *Portret proletar* | William Carlos Williams | Petre Stoica |
| 50 | *Omul care tace* | Constantin Abăluță | – |
| 51 | *Statuie* | Frank O’Hara | Constantin Abăluță |
| 52 | *Minciună?* | Mircea Ivănescu | – |
| 53 | *N–ai teamă* | Vasile Voiculescu | – |
| 54 | *Extazul* | John Donne | Ștefan Augustin Doinaș |
| 55 | *Pot să ascult* | Marta Petreu | – |
| 56 | *Când însuși glasul* | Mihai Eminescu | – |
| 57 | *Pentru că am ascultat împreună cum bătea inima micuței gimnaste în colțul covorului uriaş​* | Dan Sociu | – |
| 58 | *Lecția despre cub* | Nichita Stănescu | – |
| 59 | *Permisie totală* | Jacques Prévert | Gellu Naum |
| 60 | *Scara la cer* | Marin Sorescu | – |
| 61 | *La o–ntristare* | Ienăchiță Văcărescu | – |
| 62 | *Hai să fugim* | Nora Iuga | – |
| 63 | *Dați–mi un trup, voi munților* | Lucian Blaga | – |
| 64 | *Uvedenrode* | Ion Barbu | – |
| 65 | *Telefonul din colțul străzii* | Cristian Popescu | – |
| 66 | *Vior* | Oskar Pastior | Gabriel H. Decuble |
| 67 | *Poate că mă visează cineva* | Ana Blandiana | – |
| 68 | *Aștept pe cealaltă* | Ady Endre | George A. Petre |
| 69 | *Există* | Guillaume Apollinaire | Gellu Naum |
| 70 | *Desen atic* | Gheorghe Tomozei | – |
| 71 | *Paharul* | Ion Mureșan | – |
| 72 | *Cu majuscule* | Gheorghe Tomozei | – |
| 73 | *Lebăda cu nas* | Nina Cassian | – |
| 74 | *Rugă* | Ileana Mălăncioiu |  |
| 75 | *La marte* | Marin Sorescu | – |
| 76 | *Moartea s–a suit* | Mircea Dinescu | – |
| 77 | *Mamie, n–o să–nțelegi* | Tristan Tzara | – |
| 78 | *Domeniul presimțirilor* | Gellu Naum | – |
| 79 | *Son de Negros en Cuba* | Federico Garcia Lorca | Darie Novăceanu |
| 80 | *Tragi de–o sfoară, miști păpușa* | Charles Bukowski | Dan Sociu |
| 81 | *Boemă* | George Bacovia | – |
| 82 | *Sărutul meu* | Ady Endre | George A. Petre |
| 83 | *Întâlnire* | Ana Blandiana | – |

*Note.* The collection includes poems written originally in Romanian or other languages (e.g., English, Spanish, German, French, Italian), chosen from anthologies and covering several centuries (i.e., 16^th^ century to present time). All poems were presented in Romanian; where necessary, established translations were used.

**Supplementary Table 2.** *Correlations Between Reader Characteristics, Socio-affective and Cognitive Processes, and Emotion Intensity*

|  | **Age** | **Sex** | **Education** | **Group** | | **RE-Time** | **RE-Age** | **RE-Habits** | **TEQ** | **VVIQ** | | **VMIQ** | **BAIS-V** | **BAIS-C** | **FQ-IF** | **EMP** | **VIZ** | | **MOV** | | **WRD** | | **SND** |
| --- | --- | --- | --- | --- | --- | --- | --- | --- | --- | --- | --- | --- | --- | --- | --- | --- | --- | --- | --- | --- | --- | --- | --- |
| **Age** | - |  |  |  |  | |  |  |  |  |  | |  |  |  |  |  |  | |  | |  |  |
| **Sex** | **- .43**** | - |  |  |  | |  |  |  |  |  | |  |  |  |  |  |  | |  | |  |  |
| **Education** | **.81***** | -.27 | - |  |  | |  |  |  |  |  | |  |  |  |  |  |  | |  | |  |  |
| **RE-Time** | .27 | -.26 | .20 | - |  | |  |  |  |  |  | |  |  |  |  |  |  | |  | |  |  |
| **RE-Age** | -.12 | .21 | -.18 | -.05 | - | |  |  |  |  |  | |  |  |  |  |  |  | |  | |  |  |
| **RE-Habits** | .02 | **-.41**** | -.07 | .20 | .17 | | - |  |  |  |  | |  |  |  |  |  |  | |  | |  |  |
| **TEQ** | -.28 | .24 | -.11 | .23 | .05 | | -.07 | - |  |  |  | |  |  |  |  |  |  | |  | |  |  |
| **VVIQ** | -.06 | .08 | .10 | .18 | -.17 | | -.02 | .19 | - |  |  | |  |  |  |  |  |  | |  | |  |  |
| **VMIQ** | -.09 | .19 | .10 | -.08 | -.17 | | -02 | .26 | **.40**** | - |  | |  |  |  |  |  |  | |  | |  |  |
| **BAIS-V** | -.04 | -.18 | -.06 | .27 | -.01 | | .**38*** | .07 | .**49***** | .12 | - | |  |  |  |  |  |  | |  | |  |  |
| **BAIS-C** | -.04 | -.15 | -.04 | .28 | -.11 | | **.32*** | -.03 | **.52***** | .23 | **.75***** | | - |  |  |  |  |  | |  | |  |  |
| **FQ-IF** | -.22 | .01 | -.20 | .08 | -.13 | | .15 | .29 | **.50***** | **.37*** | **.58***** | | **.65***** | - |  |  |  |  | |  | |  |  |
| **EMP** | **- .41**** | .05 | -.28 | .02 | -.02 | | -.08 | **.36*** | .27 | .24 | **.38*** | | **.36*** | **.52***** | - |  |  |  | |  | |  |  |
| **VIZ** | -.08 | .06 | -.02 | .25 | .01 | | .04 | .26 | **.49***** | .26 | **.48**** | | **.39**** | **.50***** | **.59***** | - |  |  | |  | |  |  |
| **MOV** | .26 | .17 | .19 | .03 | .05 | | -.12 | .04 | -.21 | -.28 | -.02 | | -.20 | -.14 | .04 | .02 | - |  | |  | |  |  |
| **SOUNDS** | .01 | .19 | -.07 | .20 | .05 | | .07 | .23 | .20 | -.01 | **.33*** | | **.38*** | **.31*** | **.40**** | **.43**** | -.01 | - | |  | |  |  |
| **WORDS** | -.13 | .18 | -.08 | -.17 | .08 | | **.33*** | -.09 | .09 | -.13 | **.49**** | | **.36*** | .26 | **.41**** | **.31*** | -.08 | .29 | | - | |  |  |
| **Arousal** | **-31*** | .17 | -.12 | .10 | .01 | | .00 | .27 | **.33*** | **.48**** | **.38*** | | **.37*** | **.43***** | **.74***** | **.56***** | -.06 | .27 | | .23 | |  |  |

*Note*. Spearman’s rho. p < .001 ‘***’, p < .01 ‘**’, p < .05 ‘*’. Abbreviations: RE-Time: Reading experience – Longest period reading poetry; RE-Age: Reading experience – Age at which reading began; RE-Habits: Reading experience – Reading habits over the past six months; TEQ : Trait empathy; VVIQ: Trait visual imagery; VMIQ: Trait movement imagery; BAIS – V: Trait auditory imagery vividness; BAIS – C: Trait auditory imagery control; FQ-IF: Proneness to fantasizing; EMP: Empathy for authors and characters; VIZ: Visual imagery while reading; MOV: Movement imagery while reading; WRD: Auditory imagery for words while reading; SND: Auditory imagery for sounds while reading.
